# Improving Crops for a Changing World

**DOI:** 10.3389/fpls.2021.728328

**Published:** 2021-09-06

**Authors:** James R. Lloyd, Jens Kossmann

**Affiliations:** Department of Genetics, Institute for Plant Biotechnology, University of Stellenbosch, Stellenbosch, South Africa

**Keywords:** plant biotechnology, abiotic and biotic stress tolerance, biofuels, crop yield, functional foods, genome editing, pharming, GMO regulation

Plant biotechnology has been fundamental to the development of human civilisation. The domestication of plants helped increase food production, allowing the sustenance of populations in large settlements and they provide most calories in the human diet alongside being used as fodder for farm animals. They are also good sources of both therapeutic drugs and industrial feed stocks, while more recently they have been used to produce pharmaceutical proteins and biofuels. There are nevertheless many areas where plants can be improved through genetic manipulation and there are pressing reasons why this needs to be accomplished.

## Increasing Yield

The need for increasing crop yields to provide food for a burgeoning world population has been recognised for centuries, since at least the time of Thomas Malthus. His “An Essay on the Principle of Population” (Malthus, 1798) led to the concept of the Malthusian trap where increasing populations become starved when they outstrip growth in food production. Even though the world population has increased approximately eight-fold since 1800 to almost 8 billion in 2019 (United Nations, Department of Economic and Social Affairs, Population Division, 2019), plant breeders have managed to increase yields to keep pace with this growth and this should be celebrated as a triumph of agricultural biotechnology.

Notwithstanding this historical increase in food production there are still many issues that need to be addressed. Despite the relatively high yields of modern plant varieties, estimates indicate that 800 million people still suffer from calorie deficit (Global Nutrition Report, 2016) and this is a growing problem as the world's population is expected to reach approximately 11 billion by the end of this century (United Nations, Department of Economic and Social Affairs, Population Division, 2019). This means that agricultural yields need to continue to increase, but rates of yield increases produced by plant breeders are declining to levels that are insufficient to cope with population growth (Ray et al., 2013). In addition, anthropomorphic climate change means that the plants will have to survive with increased levels of abiotic stresses (Ray et al., 2019).

Pests are thought to be responsible for some of the highest yield decreases in crops, ranging between 25 and 40%. The highest potential losses come from weeds (34%), while insects (18%) and microbial diseases (16%) also lead to significant losses (Oerke, 2006). Some of the first commercially released transgenic crops were engineered to be resistant to broad spectrum herbicides—such as glyphosate or glufosinate ammonium—to help overcome competition. Since then their widespread use has led to the development of herbicide resistant plants with almost 40 glyphosate resistant weeds having been identified worldwide since 1996 (Heap and Duke, 2018). Although stacking herbicide resistant traits in crop plants will help reduce the development of resistance, there is still a need to identify novel herbicides alongside corresponding resistance mechanisms that can be used to expand this portfolio.

Resistance to attack by both insect and microbes can be introduced by conventional plant breeding, but loci leading to resistance can be difficult to identify and time consuming to incorporate into elite lines. Therefore, transgenic technologies are often used, especially when it comes to resisting insect predation. Expression of genes encoding insecticidal proteins from *Bacillus thuringiensis* have become the mainstay of resisting insect attack in many transgenic plants. Field developed resistance by insect pests to either Cry1 or Cry3 proteins has, however, been reported in the literature (Tabashnik, 2015) meaning that the development of crops containing improved insecticidal proteins is needed to overcome this threat to food security.

A few transgenic traits leading to resistance to microbial diseases have been commercialised, and many more have been demonstrated in model plants (Dong and Ronald, 2019). The best example of the use of transgenic technology in this respect is the Rainbow papaya which has largely replaced the conventional crop in Hawaii due to its resistance to papaya ringspot virus (Hamim et al., 2018). No natural resistance to this disease has been identified, so transgenic technology is the best method of protecting this crop. Although several other transgenic plants resistant to diseases have been approved for growth in the United States, they are not currently used. For many crops introducing genetic variation leading to disease resistance will be vital to help reduce yield penalties from microbial pathogens.

Abiotic stresses also cause major losses in crops, with estimates of more than 50%. The first transgenic crop with improved resistance to drought stress was released in 2011 and since then similar technologies have been engineered into other crops to improve abiotic stress tolerance. Tolerance in other crops is also being developed using a mixture of conventional breeding as well as transgenic and genome editing techniques and these improved plants are urgently needed given predictions of climate change leading to increased periods of abiotic stress, especially drought (McKersie, 2015).

Although alleviating crop losses by reducing biotic and abiotic stresses will greatly help increase productivity, there are other ways that yields can be increased. Many large-scale international projects are currently ongoing to accomplish this, for example the introduction of C4 type photosynthesis in rice to decrease inefficiencies caused by photorespiration (Ermakova et al., 2020) and this project, alongside others involving the rational manipulation of plant metabolism, will likely prove important in increasing food production.

## Healthier Plants

While increasing yield is important for food security, making plants healthier will help overcome nutritional deficiencies. Approximately 2 billion people currently suffer from micronutrient deficiency which can lead to stunted growth in children (Global Nutrition Report, 2020). In addition, several chronic diseases—such as type II diabetes—are influenced by the types of food that we eat.

Many biofortified plants are being developed (Garg et al., 2018) to overcome these issues through a combination of conventional breeding and transgenesis. For example, some plants contain low amounts in specific types of amino acids and populations that rely on these as staple foods can suffer from deficiencies. The development of high lysine maize by conventional plant breeding has led to large potential improvements to child health in some parts of Africa (Gunaratna et al., 2010), but increasing levels of this amino acid further would still be helpful. In addition, raising vitamin levels could help drastically improve health benefits and the recent approval of golden rice in several countries (Stokstad, 2019) is a welcome development in the fight against vitamin A deficiency that still leads to the deaths of 670,000 children per annum. Altering plants to contain increased levels of health promoting compounds such as carotenoids or omega-3 fatty acids, or to engineer the presence of compounds that act as prebiotics may also lead to health benefits.

Plants can also help in producing healthier lifestyles through the production of pharmaceutical chemicals or proteins (Chin et al., 2006; Schillberg et al., 2019). Many of the world's drugs were discovered in plants as they contain a much wider range of metabolites that many other organisms due to the specialised metabolism that they contain. Bioprospecting to identify novel pharmaceuticals, engineering genomes to increase amounts of these metabolites in plants or synthetic biology approaches to introduce such pathways in other organisms are all important methodologies that can help produce increased amounts of novel pharmaceutical. Other protein-based pharmaceuticals—such as plantibodies or vaccine epitopes—can be produced very efficiently in plants and their development and commercialisation can have a major beneficial effect on diseases in both humans and animals.

## Industrial Uses of Plants

Plants produce a number of products—such as starch and cell wall material—that can be used in large scale industrial processes. They often have to be modified before use and their modification *in planta* can, therefore, help make them more useful to for industrial uses (Zeeman et al., 2010; Loqué et al., 2015). In addition, the increase in atmospheric CO_2_ over the past century caused by the burning of fossil fuel is well-known and biofuels can help to lower the rate of increase. Currently many biofuels are produced from sugars or oils harvested from plants that could be used for food or feed and developing plants into second generation biofuel feedstocks is, therefore, imperative to try and reverse this. Second generation biofuel production is currently not economically viable due to inefficiencies in degrading plant biomass to fermentable sugars (Bhatia et al., 2017), meaning that the development of plants with more easily digested cell walls is needed. There may also be plants that are suitable for production of biofuels that can grow on non-arable land and the development of such biofuel crops is needed. The use of algae in this regard is especially interesting and the manipulation of algae metabolism to increase oil accumulation for biodiesel, or to grow faster, will likely be of great importance for future biofuels (Behera et al., 2015). Engineering plants may also allow for carbon sequestration to reverse increasing atmospheric carbon dioxide.

## Plant Biotechnology in Non-Industrialised Countries

Most commercial GM plants have been manufactured in industrialised countries and may not be suitable for growth in other parts of the world. Two examples demonstrating this come from Africa. Firstly, bollworm resistant bt cotton is no longer grown in Burkina Faso as the cotton quality from these plants was not as good as conventional local cotton varieties (Luna and Dowd-Uribe, 2020). Secondly, a study in South Africa demonstrated that locally produced maize varieties outcompeted insect resistant GM maize when there was little insect infestation (Fischer et al., 2015). These examples indicate that low- and middle-income countries will potentially miss out on the benefits of GM plants unless there is sufficient commercial incentive to engineer local varieties with this technology. This can happen either through partnerships with large agricultural biotechnology companies and/or academic institutions, or through local production.

Most calories in the human diet come from a small number of plants mainly the cereals maize, rice, wheat, millet, sorghum and some tuberous crops such as potato. There are many other plants that could act as crops if more research was applied to them and these are known as orphan crops (Ye and Fan, 2021). Examples of these include tef, yams, cassava, finger millet, pigeon pea and groundnut. These may well be more suitable for growth in non-industrialised countries, especially by small scale farmers and the development of such orphan crops would help food security.

## Changing Technologies and Their Regulation

Plant improvement encompasses several types of technologies. Until the 1990s this was dominated by marker assisted breeding, but since then transgenic and cisgenic technologies have made valuable contributions to increasing productivity (Areal et al., 2012; Klümper and Qaim, 2014). More recently various genome editing techniques have been established that can directly alter nuclear DNA (Arora and Narula, 2017; Jaganathan et al., 2018; Manghwar et al., 2019). These allow precise editing of genomes through the introduction of knockout or missense mutations at targeted loci or even the introduction of epigenetic changes. This is a fast-changing field, but the introduction of such techniques into all crop plants will be hugely helpful in allowing crop development.

Given the importance of these novel technologies to improve plant yield, regulatory processes are essential to allow development of improved crops. Many parts of the world have well-established frameworks, albeit often based on differing principles (Turnbull et al., 2021). While some parts of the world focus of trait-based approaches, others use the precautionary principle. Most developing countries, however, lack any framework which hampers their access to novel plant varieties and this is leading to a divide between industrialised and non-industrialised countries potentially increasing inequality (He and Krainer, 2021). The convergence—or further divergence—of regulatory frameworks will be critical in influencing progress of biotechnologically altered plants.

Ultimately the solution to improving crop yield will lie in a combination of different technologies including plant breeding, transgenesis, cisgenesis, genome editing, improved systems to monitor crops to assess whether they are stressed as well as better agricultural machinery. Many of these technologies have been integral in improving plant yields over the past century, while others will become increasingly important to improving plants in the future. Multidisciplinarity will be key to the successful development of these technologies to produce the crops that we will need.

The Chief Editors of the Plant Biotechnology section at Frontiers in Plant Science wish to use the section to help applied plant scientists develop improved crops. We expect that the majority of papers submitted to the Plant Biotechnology section will utilise transgenic, cisgenic or genome editing technologies and we welcome papers developing improved crop plants by those techniques as well as studies examining novel ways to improve agronomic traits in model plants. Finally, we wish to help regulatory regimes by welcoming papers discussing varying approaches to this in different parts of the world.

## Author Contributions

JRL wrote the first draft after which both authors contributed equally to the final manuscript and approved it for publication.

## Conflict of Interest

The authors declare that the research was conducted in the absence of any commercial or financial relationships that could be construed as a potential conflict of interest.

## Publisher's Note

All claims expressed in this article are solely those of the authors and do not necessarily represent those of their affiliated organizations, or those of the publisher, the editors and the reviewers. Any product that may be evaluated in this article, or claim that may be made by its manufacturer, is not guaranteed or endorsed by the publisher.
